# Cyclic AMP responsive element-binding protein promotes renal cell carcinoma proliferation probably *via* the expression of spindle and kinetochore-associated protein 2

**DOI:** 10.18632/oncotarget.7017

**Published:** 2016-01-25

**Authors:** Haihui Zhuang, Xiangyu Meng, Yanyuan Li, Xue Wang, Shuaishuai Huang, Kaitai Liu, Michael Hehir, Rong Fang, Lei Jiang, Jeff X. Zhou, Ping Wang, Yu Ren

**Affiliations:** ^1^ Zhejiang Provincial Key Laboratory of Pathophysiology, Ningbo University School of Medicine, Ningbo 315211, China; ^2^ Department of Urologic Surgery, Ningbo Urology and Nephrology Hospital, Ningbo 315000, China; ^3^ Department of Pathology, First Affiliated Hospital, Zhejiang University School of Medicine, Hangzhou 310031, China; ^4^ Laboratory of Kidney Carcinoma, Ningbo Urology and Nephrology Hospital, Urology and Nephrology Institute of Ningbo University, Ningbo 315000, China; ^5^ Ningbo Medical Center, LiHuiLi Hospital, Medical School, Ningbo University, Ningbo 315041, China

**Keywords:** CREB, SKA2, RCC, proliferation

## Abstract

Emerging evidence shows that the aberrantly expressed cyclic AMP responsive element-binding protein (CREB) is associated with tumor development and progression in several cancers. Spindle and kinetochore-associated protein 2 (SKA2) is essential for regulating the progress of mitosis. In this study, we evaluate *in vitro* and *in vivo* the functional relationship between CREB and SKA2 in renal cell carcinoma (RCC). Suppressing and replenishing CREB levels were used to manipulate SKA2 expression, observing the effects on RCC cell lines. Computational prediction and ChIP assay identified that CREB targeted *ska2* by binding its CRE sequence in the human genome. Overexpression of CREB reversed the inhibited cell growth following siSKA2 treatment, and reduced the number of cells holding in mitosis. Decreased expression of CREB suppressed RCC cell growth and xenograft tumor formation, accompanied by reduced expression of SKA2. In RCC tumor samples from patients, mRNA for SKA2 were plotted near those of CREB in each sample, with significantly increased immunohistochemical staining of higher SKA2 and CREB in the higher TNM stages. The study adds evidence that CREB, a tumor oncogene, promotes RCC proliferation. It probably achieves this by increasing SKA2 expression.

## INTRODUCTION

Cyclic AMP responsive element-binding protein (CREB) is a proto-oncogenic transcription factor [1, 2], that generally regulates various cell functions by enhancing the expression of target genes [3, 4]. Aberrant expression of CREB has been well described in non-small cell lung carcinoma [5, 6], melanoma [7], breast [8–11], hepatocellular cancers [12] and acute myeloid leukemia [13, 14]. Our previous reports showed that blocking the cyclic AMP-responsive element (CRE) site between CREB and targeted genes (Bcl-2 and cyclins), abrogated the anti-tumor drug-induced apoptosis and cell proliferation [15, 16]. It seemed that CREB played a pivotal role in promoting tumorigenesis. However, the role of CREB in renal cell carcinoma (RCC) remains less explored.

RCC ranks the third most frequent malignancy in urological oncology [17, 18]. Like other cancers, the development of RCC is a multistep process with accumulation of genetic and downstream changes [18, 19]. In order to find novel CREB-interacting proteins expressed in RCC cells, and to provide new insights into the cellular mechanisms, we identified a specific interaction between CREB and SKA2 (spindle and kinetochore-associated protein 2) by using bioinformatic software (http://mulan.dcode.org/) [20]. Although depletion of SKA2 can cause the cells to undergo a prolonged checkpoint in a metaphase-like state [21], little else is known about it.

The present studies showed that CREB is usually up-regulated in RCC tissues and cell lines. Decreased CREB expression significantly inhibited RCC cell proliferation *in vitro* and *in vivo*, accompanied by the suppression of SKA2 expression. Downregulation of SKA2 by RNAi significantly suppressed the proliferation of RCC cells and increased the cell number holding in mitosis, whereas these results could be reversed by the ectopic expression of CREB. Furthermore, analysis of clinical samples showed that the staining score of CREB was positively correlated with those of SKA2. These data suggest that CREB may function as a tumor oncogene to increase the cell proliferation by increasing expression of its target gene *ska2* in RCC.

## RESULTS

### CREB is frequently up-regulated in RCC

**Figure 1:**
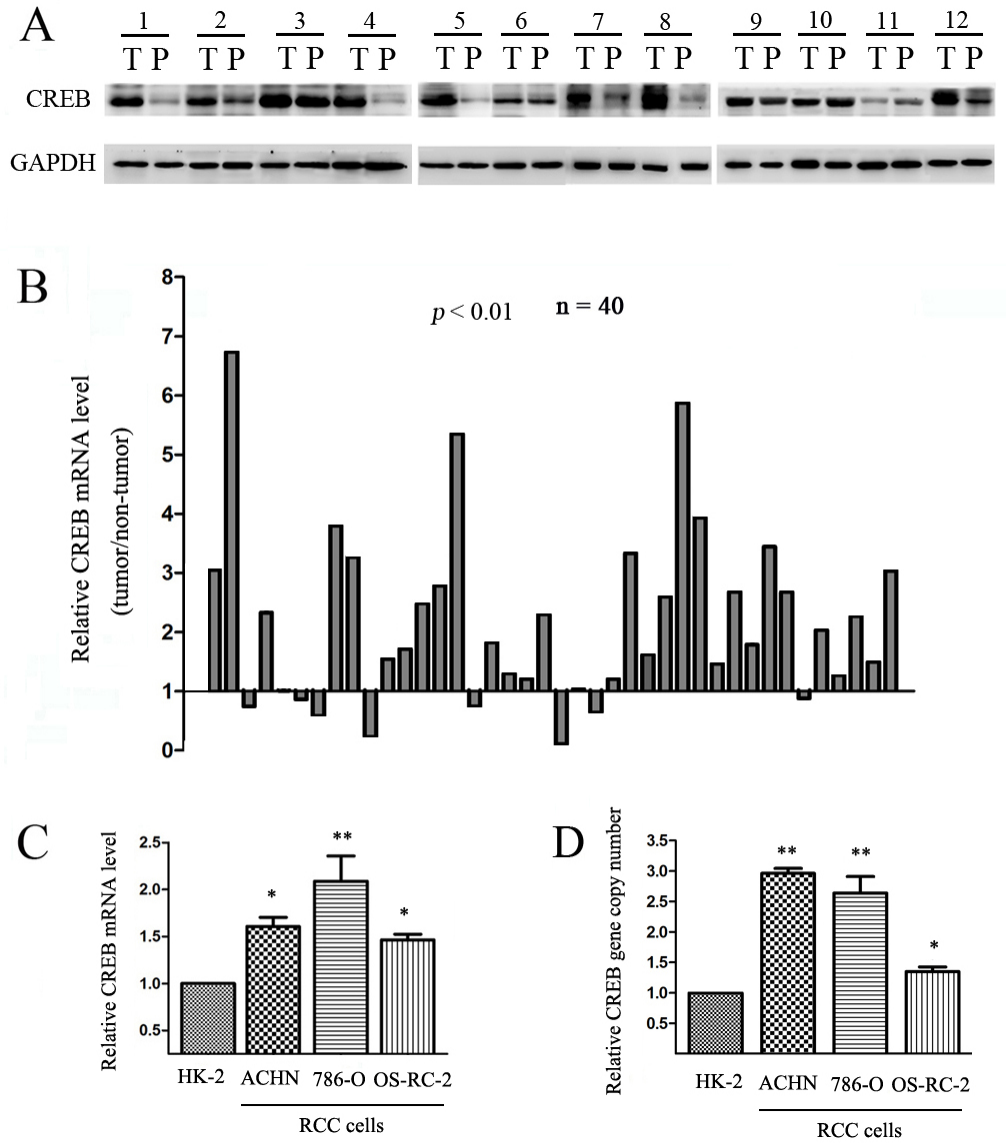
CREB expression is frequently up-regulated in RCC Relative protein expression (**A**) and mRNA level (**B**) of CREB were detected in RCC tissues (T) compared with their matched adjacent non-tumor tissues (P). In three cell lines derived from RCC (ACHN, 786-O, OS-RC-2), CREB mRNA level (**C**) and gene copy number (**D**) were also higher than nonmalignant cell line (HK-2). Data were shown as mean ± SD from three independent experiments. *P < 0.05, **P < 0.01 versus HK-2 cell.

CREB expression was initially evaluated in RCC tissues and matched adjacent non-tumor tissues. We assessed the protein expression of CREB levels by Western blot in 12 patient RCC samples, and found CREB was higher in tumor tissues than in non-tumor tissues in 9 patients (Figure 1A). Expression of CREB mRNA in RCC tissue was assessed by qRT-PCR in 40 patient RCC samples. The mRNA level of CREB was up-regulated in 30 (75%) of tumor samples (*P* < 0.05) (Figure 1B). We used qRT-PCR to examine CREB expression in ACHN, 786-O and OS-RC-2 RCC cell lines and in normal proximal tubule epithelial cell line (HK-2). CREB expression was significant higher in 100% of RCC cells than HK-2 cells (Figure 1C). Furthermore, we found amplification of the CREB gene copy number in RCC cells compared with HK-2 (Figure 1D). These findings indicate that CREB was usually overexpressed in RCC tissues and cell lines, moreover the mechanism of the up-regulation of CREB is gene copy number amplification.

### Decreased expression of CREB suppresses RCC cell growth

**Figure 2:**
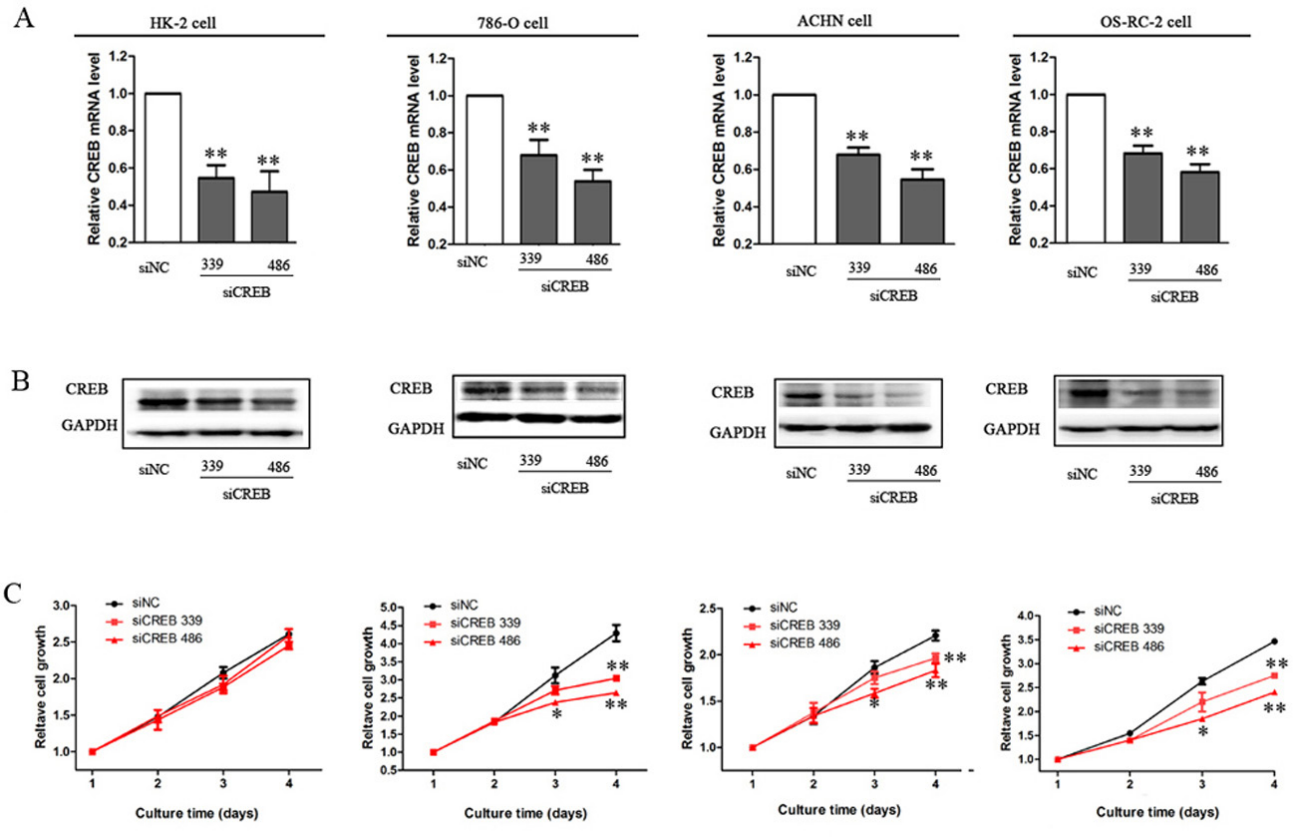
Decreased expression of CREB suppresses RCC cell growth After cells were transfected with siCREB339 or siCREB486, the mRNA level (**A**) and protein expression (**B**) of CREB were significantly decreased in HK-2, ACHN, 786-O and OS-RC-2 cells, as compared with cells transfected with siNC. Whereas, inhibition of cell proliferation was not observed in HK-2 cell, but in RCC cells (**C**). Data were shown as mean ± SD from three independent experiments. *P < 0.05, **P < 0.01 versus siNC.

To explore the role of CREB in RCC cell proliferation, we down-regulated CREB expression to investigate the effect on cell proliferation *in vitro*. After transfecting specific CREB siRNA (339 and 486) into RCC cell lines and HK-2 cells, the mRNA and protein levels of CREB were significantly decreased, furthermore the efficacy of siCREB486 was greater than that of siCREB339 (Figure 2A, 2B). RCC cell proliferations were also significantly reduced, whereas, no effect was shown in HK-2 cells (*P* < 0.05, Figure 2C). We have previously reported that decreasing the pCREB inhibits the growth of RCC, by dominant negative CREB mutation in which the serine residue at 133 was replaced with threonine [22]. Here, we used two siRNA types to decrease the CREB expression and cause a significant inhibition of RCC cell proliferation. These results produce stronger evidence *in vitro* that suppression of CREB expression inhibits RCC cell proliferation.

### SKA2 is a CREB targeted gene

**Figure 3:**
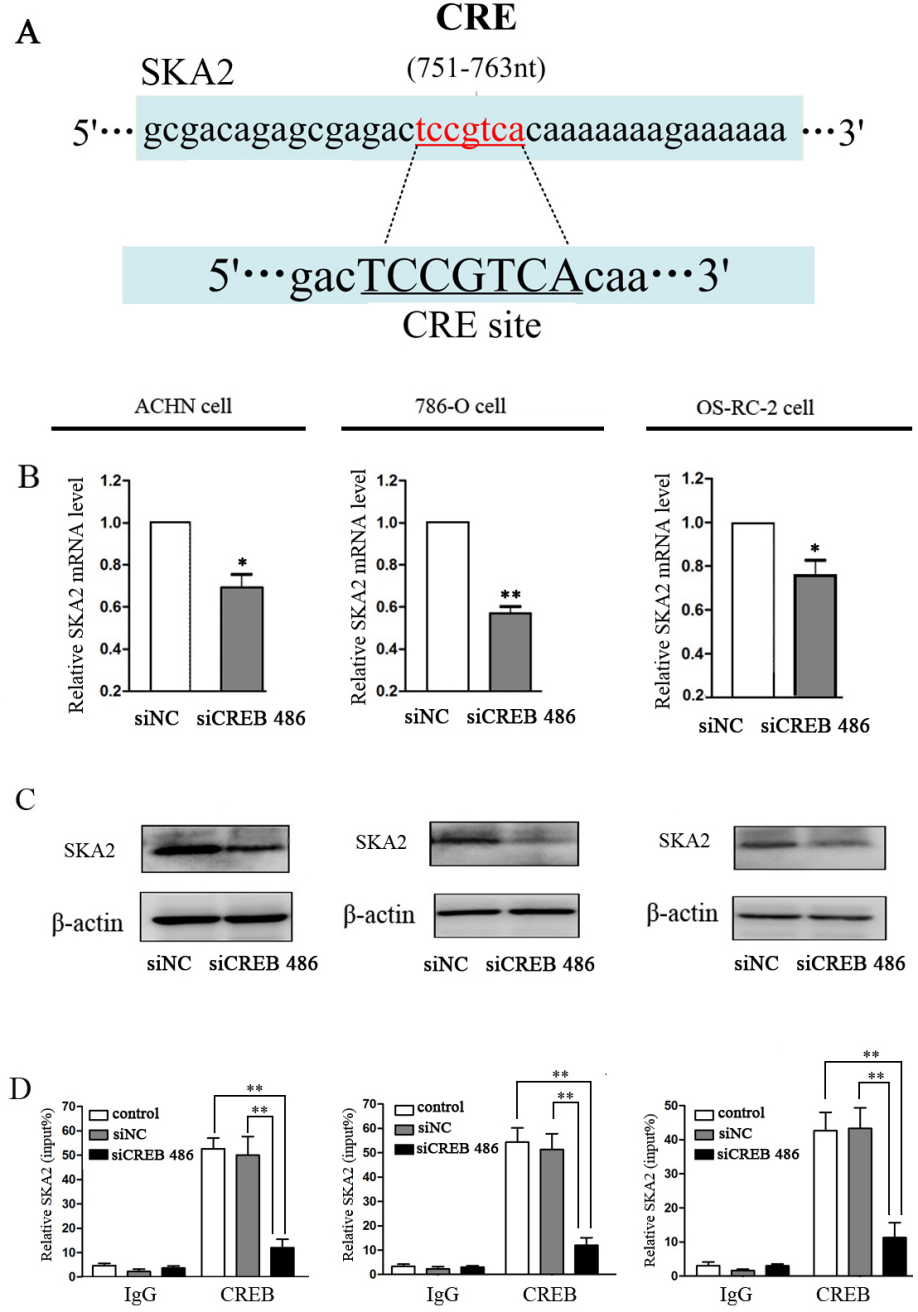
SKA2 is a CREB targeted gene The potential CREB targeting site in SKA2 promoter, as detected by MultiTF tools (**A**). After down-regulation of CREB with siCREB, the mRNA (**B**) and protein (**C**) levels of SKA2 were significantly reduced in RCC cells. CHIP with specific CREB antibody showed binding of CREB to the putative promoter of SKA2 in RCC cell lines (**D**). Data were shown as mean ± SD from three independent experiments. *P < 0.05, **P < 0.01 versus siNC treatment.

To investigate the mechanism of CREB function in RCC carcinogenesis, we employed MultiTF tools (http://mulan.dcode.org/) to look for putative human protein-coding gene targets of CREB. The gene *ska2,* which has the CRE site, was further studied as a potential target (Figure 3A). Down-regulation of CREB greatly reduced the mRNA and protein levels of SKA2 in ACHN, 786-O and OS-RC-2 cells (Figure 3B, 3C). ChIP assay also revealed that CREB bound to the promoter of SKA2 and increased protein expression in RCC cells (Figure 3D).

### SKA2 is involved in the CREB-regulated cell proliferation *in vitro*

**Figure 4:**
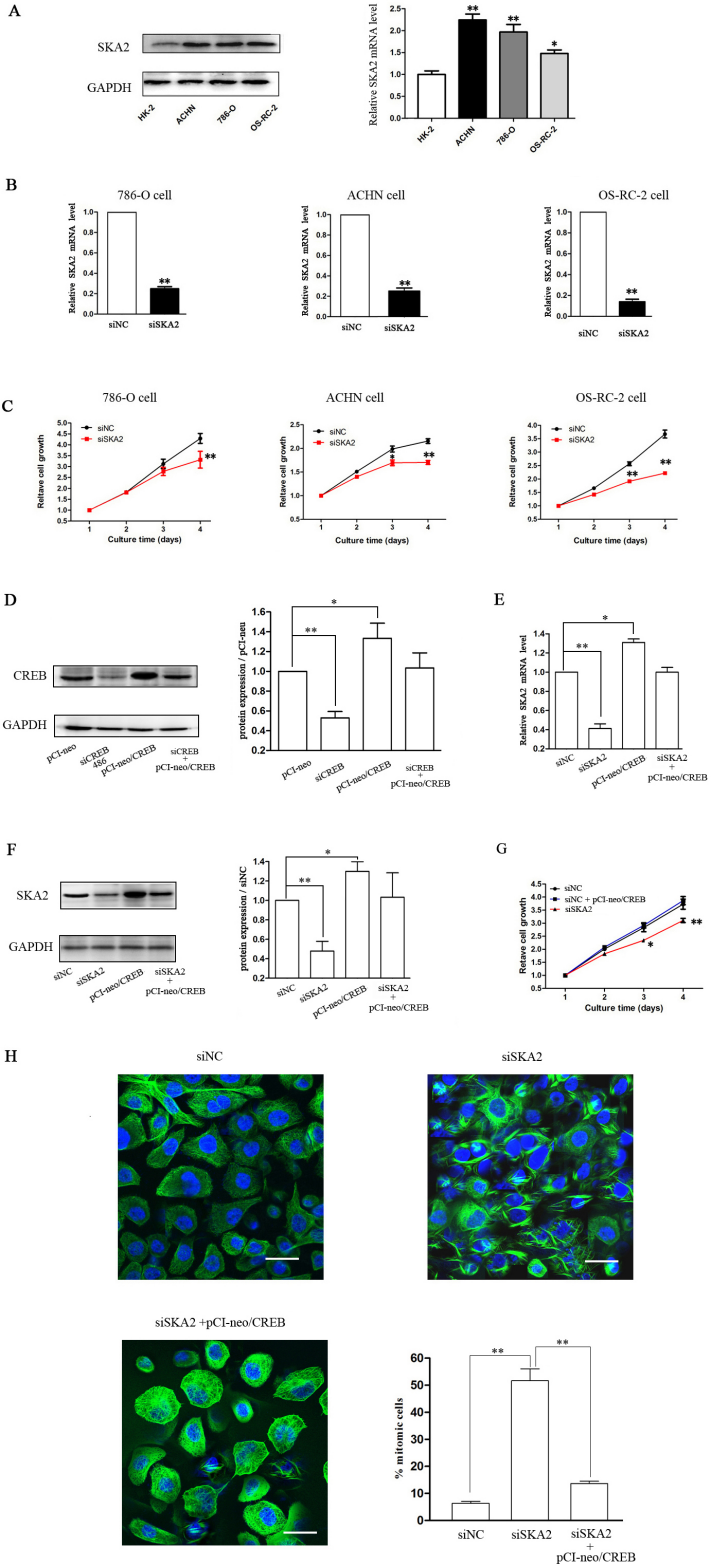
SKA2 is involved in CREB-regulated cell proliferation The mRNA and protein levels of SKA2 in RCC cells were significantly higher than HK-2 cell (**A**). After cells were transfected with siSKA2, the mRNA levels were significantly decreased (**B**), cell proliferation was inhibited (**C**). However, overexpression of CREB in OS-RC-2 cells co-transfected with pCI-neo/CREB (**D**), reversed siSKA2-reduced mRNA level (**E**), protein levels (**F**), cell growth (**G**) and cell division (**H**). Data are shown as mean ± SD from three independent experiments. *P < 0.05, **P < 0.01 versus HK-2 cell or siNC treatment. Scale bar, 10 μm.

To explore whether SKA2 was involved in CREB-regulated cell proliferation, we first explored the role of SKA2 in RCC growth, and found that SKA2 mRNA and protein levels in RCC cells were significantly higher than in HK-2 cells (Figure 4A). After successfully decreasing SKA2 mRNA levels (Figure 4B) by siSKA2 treatment, RCC cell proliferation was inhibited (Figure 4C). These data indicated that SKA2 could also independently influence tumor cell proliferation *in vitro*.

In addition, using pCI-neo/CREB vector containing the CREB coding sequence [23] increased the expression of CREB (Figure 4D) in OS-RC-2 RCC cells, to examine whether overexpression of CREB could counteract the reduction of SKA2 and the inhibition of cell proliferation. The result was that ectopic expression of CREB reversed the suppression of SKA2 mRNA (Figure 4E) and protein (Figure 4F), and reversed the decrease of cell proliferation (Figure 4G). In addition, siSKA2-treatment significantly increased the cell number holding in mitosis, whereas this was reversed by the combined treatment with siSKA2 and pCI-neo/CREB (Figure 4H).

Taken together, these results are consistent with our hypothesis that CREB probably promotes RCC cell proliferation by increasing SKA2 expression.

### Down-regulation of CREB suppresses xenograft tumor formation and reduces SKA2 expression *in vivo*

**Figure 5:**
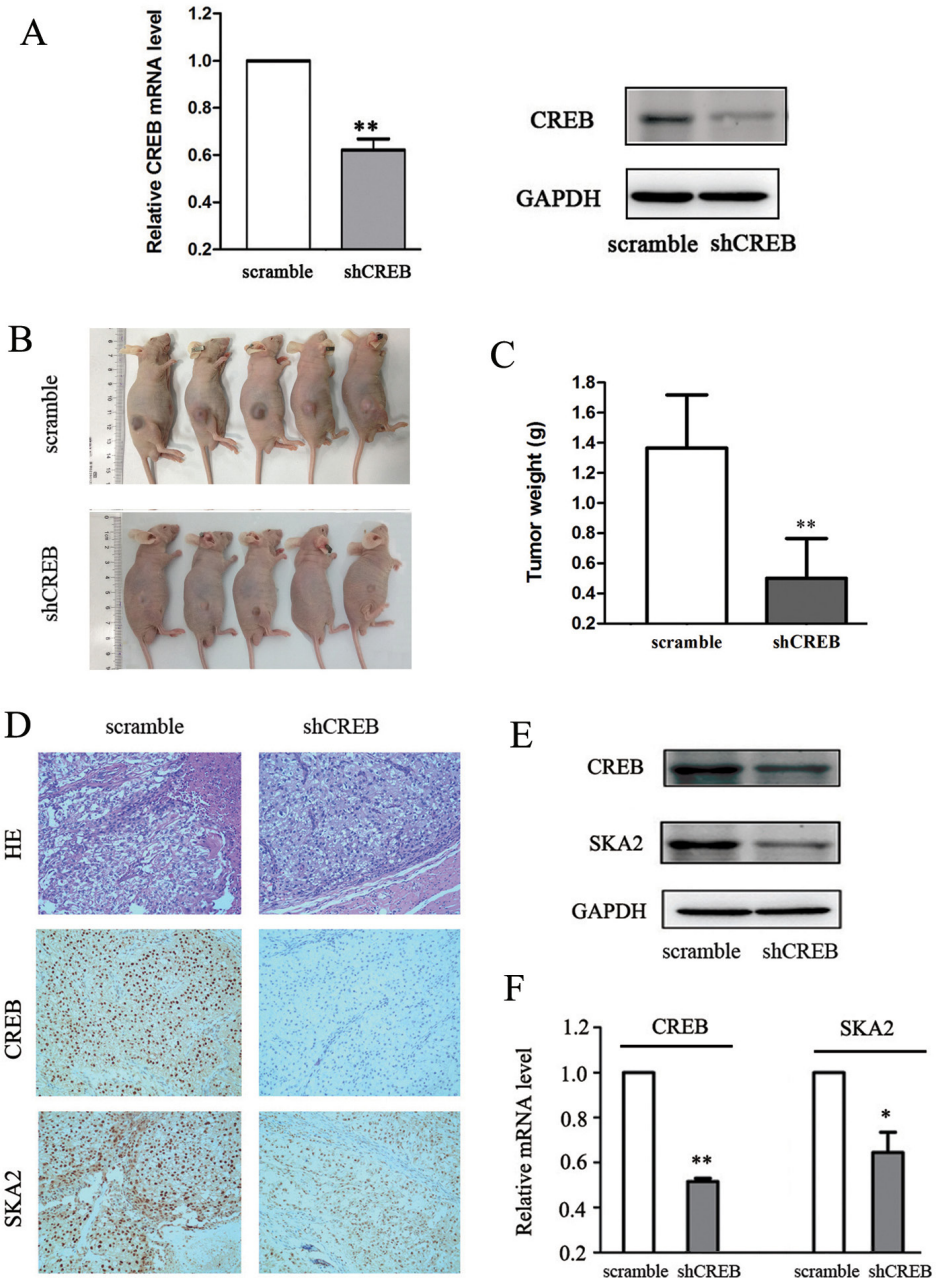
Down-regulation of CREB suppresses xenograft tumor formation and reduces SKA2 expression *in vivo* Specific shCREB treatment induced a significant decrease of CREB mRNA and protein levels in OS-RC-2 cells (**A**). Smaller xenograft tumor formation was shown in shCREB treatment (**B**), and the average weights of tumors was lower (**C**) as compared with nude mice injected with OS-RC-2 cells containing scramble. IHC staining showed that the staining score for SKA2 was also lower than in controls (**D**). Furthermore, the SKA2 protein expression (**E**) and mRNA level (**F**) was significantly decreased compared with controls. Data are shown as mean ± SD. *P < 0.05, **P < 0.01 versus scramble. Representative images were captured at a magnification of × 200.

To further evaluate the potential effect of CREB on RCC growth *in vivo*, OS-RC-2 cells were stablely transfected with scramble or shCREB by lentiviral plasmids and subcutaneously injected into nude mice. ShCREB treatment significantly decreased CREB expression levels in OS-RC-2 cells at both mRNA and protein levels (Figure 5A). 30 days after tumor cells transplantation, downregulated CREB led to a profound suppression of tumor growth, shown by a significant reduction in tumor volume and weight (Figure 5B, 5C). These data suggested that CREB inhibition decreased xenograft tumor formation of RCC cells *in vivo*.

To determine whether SKA2 was regulated by the effect of CREB *in vivo*, we tested SKA2 protein and mRNA levels in tumor tissues isolated from the mice which injected with OS-RC-2 cells containing shCREB or scramble. Weaker nuclear staining for SKA2 was shown in shCREB group than scramble group (Figure 5D). The distribution of staining scores for the tumor tissue was counted. In the scramble group, 4 mice had a score of 3 + and 1 mice had a score of 1 + ; meanwhile 2 mice had a score of 2 + and 3 mice had a score of 1 + in the shCREB group. Furthermore, Western blot analysis and qRT-PCR indicated that decreasing levels of SKA2 was accompanied by CREB knockdown (Figure 5E, 5F). These data suggested that CREB knockdown can inhibition SKA2 expression *in vivo*.

### The association of CREB with SKA2 levels in RCC tissues

**Figure 6:**
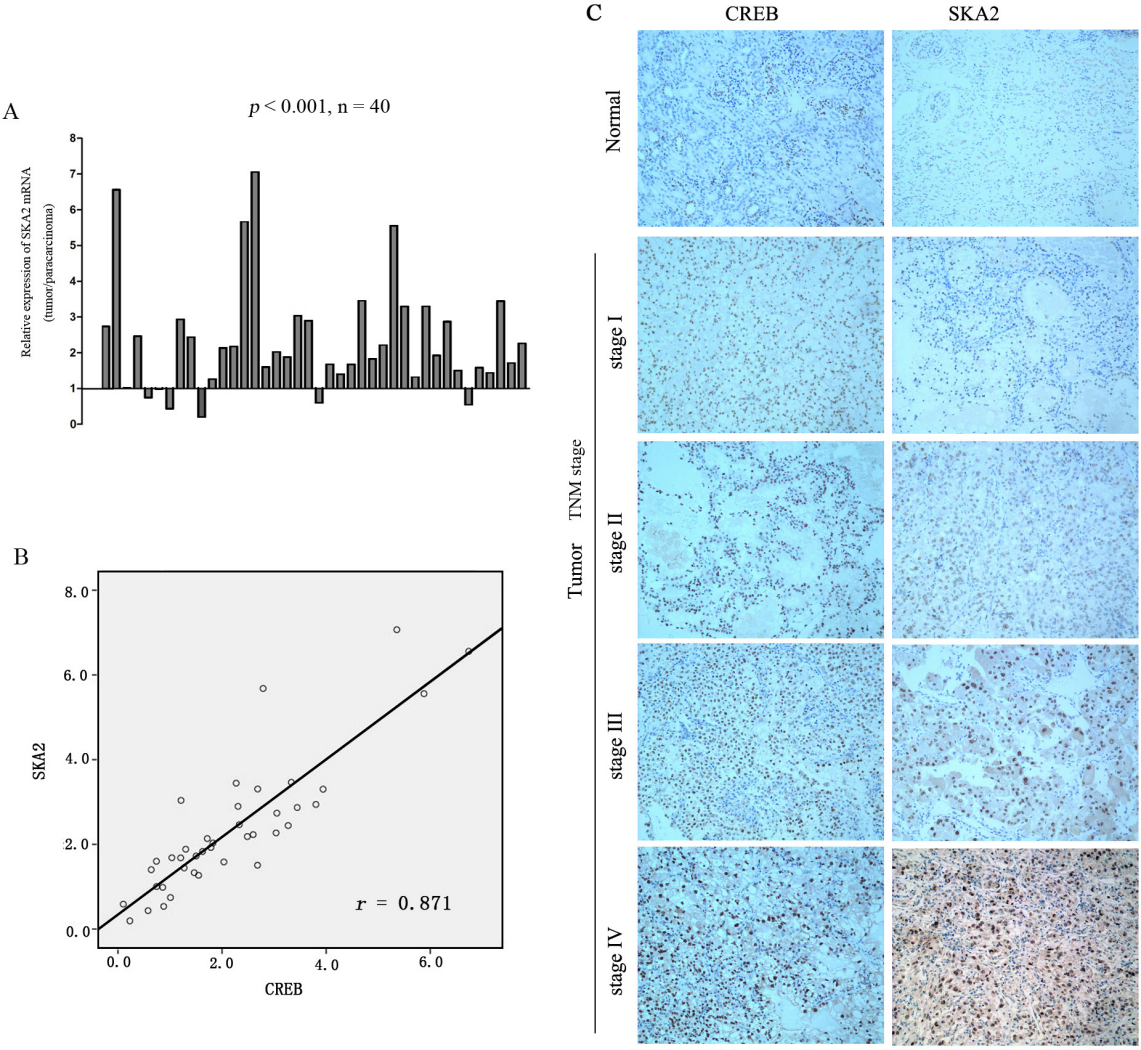
The correlation between SKA2 and CREB expression in RCC tissue sample The mRNA level of SKA2 was up-regulated in RCC cancers compared to their matched adjacent non-tumor tissues (**A**). Positive correlation between the mRNA levels of CREB and SKA2 in RCC cancers versus their matched non-tumor counterparts. A statistically significant correlation between CREB expression and SKA2 protein was observed by Pearson's correlation coefficient (**B**). Representative immunohistochemical staining of higher CREB is concomitant with higher SKA2 in tumor, but not in benign tissues, from the same patient, with increasing expression seen at higher TNM stages from I-IV (*n* = 166) (Figure C). Representative images were captured at a magnification of × 200.

Since we have shown that CREB was frequently increased in RCC and suggested targeting of SKA2 to increase cell proliferation, we investigated whether SKA2 expression level is associated with the malignancy in clinical samples. The results revealed that SKA2 mRNA level was significantly up-regulated in RCC tissues compared to their matched adjacent non-tumor tissues (Figure 6A). In serial sections of the same patient's tumor, the relative mRNA levels of SKA2 were plotted near that of CREB in each patient, a significant positive correlation was found (*p* < 0.05; *r* = 0.871) (Figure 6B).

In order to ascertain the clinical relevance of these findings, we analyzed CREB and SKA2 immunohistochemical staining in RCC specimens (*n* = 166). Intense signals of CREB and SKA2 were observed in the nucleus of tumor cells in TNM stages III and IV RCC tissues, more than those in stages I and II. Very little was seen in the non tumor tissues (Figure 6C). Significant positive correlations were seen between CREB and SKA2 immunostaining in the RCC specimens (*p* = 0.001, *r* = 0.7) (Table 1). These results provided visual corroboration of overexpression of both CREB and SKA2 in RCC tissues, and indicated that CREB overexpression may be associated with the increase of SKA2 protein levels in RCC at increasing TNM stages.

**Table 1:** CREB immunohistochemical positive correlates with SKA2 in RCC samples (n = 166)

CREB	SKA2		Total
	Low	High	
Low	36	9	45
High	11	110	121
Total	47	119	166

*P* < 0.0001, Fisher's exact test.

*r* = 0.7; *r*, Spearman correlation coefficient.

## DISCUSSION

CREB has been reported to have an impact on carcinogenesis [1, 24]. In the present study, CREB expression is substantially up-regulated in RCC tissues and cell lines according to the results of Western-blot and qRT-PCR (Figure 1). This is consistent with the results in acute myeloid leukemia [25, 26], glioma [27, 28] and breast cancer [29].

To move on from the concept of CREB expression and increasing proliferation in RCC, we examined targets of CREB using Multi TF tools software (Figure 3A). Having identified SKA2 as a target of CREB, we then discovered the binding of CREB to the promoter sequence of SKA2 using CHIP analysis (Figure 3D). This link has previously been established in lung cancer but not in RCC [30]. Our laboratory and clinical sample studies were then designed to show cause and effect in this developing concept.

We confirmed that CREB expression is increased in ACHN, 786-O and OS-RC-2 RCC cell lines *in vitro* compared to benign renal tubular HK-2 lines (Figure 1). These data are in line with our previous report regarding pCREB and data from previous studies [22]. *In vitro* studies using 2 separate transfected siCREB cell lines showed reduced proliferation compared to benign HK-2 controls (Figure 2). We also confirmed that CREB silencing significantly decreased SKA2 expression (Figure 3B, 3C). These studies reinforce the concept in previous reports from our group using pCREB mutation and other publications [2, 22].

SKA2 protein is shown to have a role in regulating progression through mitosis [21], knockdown of SKA2 produced a prolonged checkpoint dependent delay in a metaphase-like state in HeLa and A549 cells [31, 32]. Our Western-blot and qRT-PCR studies of SKA2 levels in RCC cell lines showed increased expression compared to HK-2. RCC cell line proliferation *in vitro* was reduced with siSKA2, and this effect was reversed by ectopic administration of CREB, also decreasing the siSKA2 effect of holding numbers of cells in mitosis (Figure 4). To our best knowledge, the *in vitro* suppression of RCC cell line proliferation by siSKA2 treatment is shown for the first time by our studies as well as the observation of depleted SKA2 holding RCC cells in mitosis.

Our *in vivo* data showed that when compared to control, shCREB-treated OS-RC-2 cells achieved reduced tumor growth after 30 days in nude mice. The SKA2 levels in these tumors were reduced in line with reduced tumor growth. This, to our knowledge, is the first report of the association between shCREB and reduced *in vivo* RCC cell line proliferation accompanied by SKA2 depletion. Our report also seems to be the first to show *in vivo* link of these effects in any cancer type (Figure 5). Our clinical data shows a strong correlation between nuclear staining of both CREB and SKA2 in RCC cell nuclei. Interesting increases in both CREB and SKA expression were noted in higher TNM stages (Figure 6). From a theoretical basis, knowing the effect of CREB on RCC proliferation, the link with SKA2 is proposed following Genome study. *In vitro* and *in vivo* evidence of this link is presented. Corroboration data from clinical samples is provided. Our data support the oncogene effect of CREB mediated by SKA2 in RCC.

While we show convincing data in support of our hypothesis, there are weaknesses in our study as follows: luciferase reporter assays will be helpful to explore the binding CREB to 3′-UTR of SKA2. As yet, we do not have explanations for the clinical RCC samples which did not show up-regulation of CREB. As RCC includes a number of dominant cell types and presumably many genetic mutation and tumorogenesis pathways, the subject is in its infancy and requires vigorous further research. When ectopically administered pCI-neo/CREB appeared to reverse the inhibition of proliferation caused by siSKA2, the mechanism was not fully clear. The *in vivo* work showing reduction of tumor growth, paralleled with lower SKA2 expression, merits further corroboration with statistically significant data.

## CONCLUSIONS

The present study indicated that CREB functions as tumor oncogene, promoting renal cell cancer proliferation, probably by upgrading SKA2 expression.

## MATERIALS AND METHODS

### Tissue samples

Tumor specimens and adjacent non-tumor renal tissues were collected from the Pathology Department, Ningbo Urology and Nephrology Hospital. All specimens were obtained on the basis of their availability for research purposes and under a protocol approved by the local medical ethics committee of Ningbo Urology and Nephrology Hospital. Written consent was obtained from the patients prior to their participation in the study. The tumors were classified according to the 2009 American Joint Commission for Cancer Staging TNM classification. The clinicopathological characteristics of included patients are summarized in Table 2.

**Table 2:** Clinical and pathological parameters of patients

Characteristics of tumor tissues	No. of Patients
All patients	166
Gender	
Male	90
Female	76
Age (mean (minimum-maximum))	
Male	52 (30–78)
Female	51 (38–76)
Classificationof the Common Histological Subtypes	
Clear Cell Renal Cell Carcinoma	150
Papillary Renal Cell Carcinoma	7
Chromophobe Renal Cell Carcinoma	9
Differentiation	
Well	45
Moderate	88
Low	33
Invasion depth	
T1	68
T2	53
T3	27
T4	18
Lymph node metastasis	
N0	55
N1	64
N2	35
N3	12
Distant metastasis	
M0	142
Ml	24
TNM stage	
I	35
II	50
III	46
IV	35

### Cell culture and transfections

Human renal cell carcinoma (RCC) cell lines (786-O, ACHN, OS-RC-2), an immortalized proximal tubule epithelial cell line (HK-2), and HEK-293T were purchased from American Type Culture Collection (ATCC, Shanghai, China). The 786-O and OS-RC-2 were cultured in RPMI 1640 medium (HyClone, UT, USA), ACHN, HK-2 and HEK-293T were cultured in DMEM (HyClone, UT, USA) medium, and supplemented with 10% fetal bovine serum (FBS, ExCell Bio, Shanghai, China). All cell cultures were carried out in a humidified chamber at 37°C with an atmosphere of 5% CO_2_.

siRNA for CREB: siCREB (sence: 5′-GUCUCCAC AAGUCCAAACATT- 3′, antisence: 5′-UGUUUGGACU UGUGGAGACTT- 3′), siRNA for SKA2: siSKA2 (sence: 5′-GGCUGGAAUAUGAAAUCAATT- 3′, antisence: 5′-UUGAUUUCAUAUUCCAGCCTT- 3′) and siRNA for negative control: siNC (sence: 5′-UCCUCCGAACGUG UCACGUTT- 3′, antisence: 5′-ACGUGACACGUUC GGAGAATT- 3′) were purchased from GenePharma (Shanghai, China). Transfection of siCREB, siSKA2 or siNC were transfected using the Lipofectamine 2000 transfection reagent (Invitrogen, USA) following the manufacturer's instructions [33].

### Cell proliferation assay

Cell proliferation was examined using CellTiter96^®^ AQueous One Solution Cell Proliferation Assay (Promega, WI, USA). Briefly, cells transfected with siCREB, siSKA2 or siNC were plated in a 96-well tissue culture plate. Following incubation for 24, 48, 72, and 96 h, CellTiter 96^®^ AQueous One Solution was added to each well and the cells were incubated for another 3 h at 37°C. The absorbance at 490 nm was measured using a spectrophotometer [34].

### Western blot analysis

Cells were lysed and protein was harvested with RIPA buffer (solarbio, Beijing, China) and quantified by bicinchoninic acid (BCA) analysis (Beyotime, Shanghai, China). The proteins (50 μg) were then separated on 12% SDS/PAGE gel and then transferred onto PVDF membranes (Millipore, Billerica, MA). After 5% BSA blocking, membranes were incubated with appropriate dilutions of specific primary antibodies overnight at 4°C [35]. The following antibodies were used: anti-CREB, GAPDH (Cell Signaling Technology, MA, USA) and anti-SKA2 antibody (Abcam, Cambridge, UK). Then, membranes were incubated with horseradish peroxidase-labeled secondary antibody (Boster, Wuhan, China). The protein bands were visualized using enhanced chemiluminescence reagent.

### DNA, RNA preparation, reverse transcription, gene copy number and quantitative real-time RT-PCR (qRT-PCR) analysis

Genomic DNA from the RCC cells were extracted using the Tissue DNA kit(Omega, Norcross, GA, USA). Total RNA was isolated from cells using the Trizol reagent (Invitrogen, USA). The amounts of total RNA were quantified using spectrophotometric measurements. cDNA was prepared using a reverse transcription kit (Thermo, USA). Quantitative realtime PCR (qRT-PCR) was performed in duplicate with the fluorescent DNA binding dye SYBR green (Roche, US) to detect the gene copy number and mRNA expression with special primers (Table 3) [36].

**Table 3:** Primer sequences of CREB and SKA2 for qRT-PCR and ChIP assays

Gene	Primer sequences
CREB	F: 5′-GAAGATTCACAGGAGTCAGTGGATA-3′ R: 5′-GAAGATTCACAGGAGTCAGTGGATA-3′
gCREB	F: 5′-GTCATGGCCTACGAGGAGAA-3′ R: 5′-CACGTCAGGGAGAAGCAGAG-3′
SKA2	FI: 5′-CTGAAACTATGCTAAGTGGGGGAG -3′ R1: 5′-TTCCAAACATCCTGACACTCAAAAG-3′
SKA2 (for CHIP)	F2: 5′-GTACACACGCATGCACACAC-3′ R2: 5′-AGGCAAGGGAAATAGGGAAA-3′
GAPAD	F: 5′-AAGCCTGCCGGTGACTAAC-3′ R: 5′-GCATCACCCGGAGGAGAAAT-3′
gGAPAD	F: 5′-GGTCATCCATGACAACTTTGG-3′ R: 5′-GGCCATCACGCCACAG-3′

### Chromatin immunoprecipitation (ChIP)

For ChIP assay, approximately 1 × 10^7^ cells were harvested in medium and fixed with 1% formaldehyde. Glycine solution was added at a final concentration of 0.125 M to quench unreacted formaldehyde. Fixed cells were collected by spinning at 1500 rpm for 5 min. ChIP assays were performed using a ChIP Assay Kit (Cell Signaling Technology, Inc, USA) according to the manufacturer's instructions. IgG antibody was from the ChIP Assay Kit. CREB antibody was obtained from (Cell Signaling Technology, Inc, USA). Quantification of immunoprecipitated DNA was performed using qRT-PCR with LightCycler 480 SYBR Green I Master (Roche, US). The primers shown in Table 3 were designed according to the promoter region of SKA2 gene. Values derived from three independent experiments were normalized by background signals (IgG) and presented as percentage of Input chromatin (% Input) [37, 38].

### Xenograft model in nude mice

Five-week-old male nude mice (BALB/C) (Shanghai laboratory animal center, China) were used for xenograft model with a protocol approved by the Institutional Animal Ethics Committee of Ningbo University. 3 × 10^6^ OS-RC-2 cells expressing shCREB or scramble shRNA were injected subcutaneously into the flank of mouse (*n* = 5 for each group). Every effort was made to minimize animal suffering. Thirty days after tumor cells inoculation, the mice were sacrificed. The tumors were weighed, lysed for protein and mRNA preparations (analysed by Western blot and qRT-PCR) and prepared for IHC. The results of IHC were scored in a semiquantitative manner as previously reported [22].

### IHC staining and evaluation

Immunohistochemical (IHC) analysis was performed on formalin-fixed paraffin-embedded tissue sections (5 μm thickness) of RCC tumors both from nude mice model and from clinical samples. After dewaxing and rehydration, sections were treated with 10% H_2_O_2_. After 5% BSA blocking, all sections were incubated with the primary antibodies at 4°C overnight. The primary antibodies used were: anti-CREB (Cell Signaling Technology, MA, USA) and anti-SKA2 (Abcam, Cambridge, UK) antibodies were diluted at 1:500. The secondary antibody was horseradish peroxidase-goat anti-rabbit IgG (Zhongshan Jinqiao, Beijing, China). Diaminobenzidine was used to develop the signal. Sections were lightly counterstained with hematoxylin [39].

### Fluorescent immunocytochemistry

After cells had grown on poly-L-lysine (PLL)-coated coverslips for 24 h, cells were fixed with 4% (w/v) paraformaldehyde and 0.5% Glutaraldehyde in PBS for 5 min, and then permeabilized with 0.1% Triton X-100 in PBS at room temperature for 15 min. Nonspecific antibody binding was blocked by incubation in blocking buffer, containing 1% BSA at room temperature for 60 min. Cells were incubated with β-tubulin rabbit mAb conjugated to Alexa Flour 488 (1:500, Cell signaling Technology, MA, USA) in 1% BSA overnight, DAPI fluorescent dye 1 μg/ml, (Sigma, St Louis, MO, USA). Finally, coverslips were washed twice with PBS and photographed using confocal scanning microscopy [40].

### Lentiviral vector production

Lentiviral plasmids containing optimized CREB short hairpin (shRNA) or scramble sequences were produced using 293T cells, and were used to infect OS-RC-2 cells. The CREB-specific shRNA and scrambled control sequences shRNA were designed using the RNAi Consortium tool (http://www.broadinstitute.org/rnai/trc). The sequences for CREB shRNAs were ACAGCACCCACTAGCACTATT. The sequence for scramble control was CCTAAGGTTAAGTCGCCCTCG. Double-stranded oligonucleotides representing the complementary sequences separated by a hairpin loop were cloned into pLKO.1 puro plasmids. Three plasmids pLKO.1 puro, pCMV-dR8.2 dvpr and pCMV-VSVG were transfected into HEK-293T cells. Lentivirus-containing supernatants were harvested 72 h after transfection and filtered using 0.45 μm cellulose acetate filters, and used to infect OS-RC-2 cells. Subsequently, cells were selected for positive clones using puromycin.

### Statistical analysis

All experiments were repeated three times, except for the mouse cohorts examined in 2 cohorts. The data were expressed as means ± SD. Statistical analyses were performed with SPSS software. Differences were considered significant if *P* < 0.05.
